# The prognostic value of the SOFA score in patients with COVID-19: A retrospective, observational study

**DOI:** 10.1097/MD.0000000000026900

**Published:** 2021-08-13

**Authors:** Zheng Yang, Qinming Hu, Fei Huang, Shouxin Xiong, Yi Sun

**Affiliations:** a Department of Infectious Disease, Jingzhou Hospital, Yangtze University, Jingzhou, China; b Medical Department, Jingzhou Hospital, Yangtze University, Jingzhou, China; c Department of Dermatology, Jingzhou Hospital, Yangtze University, Jingzhou, China.

**Keywords:** ARDS, COVID-19, prognosis, SARS-CoV-2, severity, SOFA score

## Abstract

Coronavirus disease 2019 (COVID-19) can lead to serious illness and death, and thus, it is particularly important to predict the severity and prognosis of COVID-19. The Sequential Organ Failure Assessment (SOFA) score has been used to predict the clinical outcomes of patients with multiple organ failure requiring intensive care. Therefore, we retrospectively analyzed the clinical characteristics, risk factors, and relationship between the SOFA score and the prognosis of COVID-19 patients.

We retrospectively included all patients ≥18 years old who were diagnosed with COVID-19 in the laboratory continuously admitted to Jingzhou Central Hospital from January 16, 2020 to March 23, 2020. The demographic, clinical manifestations, complications, laboratory results, and clinical outcomes of patients infected with the severe acute respiratory syndrome coronavirus-2 were collected and analyzed. Clinical variables were compared between patients with mild and severe COVID-19. Univariate and multivariate logistic regression analyses were performed to identify the risk factors for severe COVID-19. The Cox proportional hazards model was used to analyze risk factors for hospital-related death. Survival analysis was performed by the Kaplan–Meier method, and survival differences were assessed by the log-rank test. Receiver operating characteristic (ROC) curves of the SOFA score in different situations were drawn, and the area under the ROC curve was calculated.

A total of 117 patients with confirmed diagnoses of COVID-19 were retrospectively analyzed, of which 108 patients were discharged and 9 patients died. The median age of the patients was 50.0 years old (interquartile range [IQR], 35.5–62.0). 63 patients had comorbidities, of which hypertension (27.4%) was the most frequent comorbidities, followed by diabetes (8.5%), stroke (4.3%), coronary heart disease (3.4%), and chronic liver disease (3.4%). The most common symptoms upon admission were fever (82.9%) and dry cough (70.1%). Regression analysis showed that high SOFA scores, advanced age, and hypertension were associated with severe COVID-19. The median SOFA score of all patients was 2 (IQR, 1–3). Patients with severe COVID-19 exhibited a significantly higher SOFA score than patients with mild COVID-19 (3 [IQR, 2–4] vs 1 [IQR, 0–1]; *P* *<* .001). The SOFA score can better identify severe COVID-19, with an odds ratio of 5.851 (95% CI: 3.044–11.245; *P* < .001). The area under the ROC curve (AUC) was used to evaluate the diagnostic accuracy of the SOFA score in predicting severe COVID-19 (cutoff value = 2; AUC = 0.908 [95% CI: 0.857–0.960]; sensitivity: 85.20%; specificity: 80.40%) and the risk of death in COVID-19 patients (cutoff value = 5; AUC = 0.995 [95% CI: 0.985–1.000]; sensitivity: 100.00%; specificity: 95.40%). Regarding the 60-day mortality rates of patients in the 2 groups classified by the optimal cutoff value of the SOFA score (5), patients in the high SOFA score group (SOFA score ≥5) had a significantly greater risk of death than those in the low SOFA score group (SOFA score < 5).

The SOFA score could be used to evaluate the severity and 60-day mortality of COVID-19. The SOFA score may be an independent risk factor for in-hospital death.

## 1 Introduction

The novel coronavirus disease 2019 was caused by severe acute respiratory syndrome coronavirus-2 (SARS-CoV-2), which was termed coronavirus disease 2019 (COVID-19) on February 11.^[1]^ SARS-CoV-2 can simultaneously infect ciliated cells and secretory cells of the human respiratory epithelium. Therefore, compared with common coronaviruses, it is more infectious and pathogenic.^[2]^ As of June 10, 2021 (10:59 am CEST), the World Health Organization website reported that the number of confirmed infections worldwide reached 173,989,093, including 3,756,947 deaths and 216 countries or regions infected.^[1]^ The number of deaths caused by the COVID-19 epidemic may be greater than officially reported.^[3]^ The world is currently in the midst of a COVID-19 pandemic. Patients with COVID-19 may be clinically asymptomatic, but severe patients may have poor clinical prognosis and may experience acute respiratory distress syndrome, organ dysfunction, shock, acute kidney injury, acute heart injury, or even death.^[4]^ In the early series of cases in Wuhan, China, 26% of patients were admitted to the intensive care unit (ICU), with a mortality rate of 4.3%.^[5]^ Therefore, reliable prognostic indicators are greatly needed because they can provide an accurate evaluation of the disease and aid in the selection of more effective treatment strategies. However, effective and simple methods to evaluate the severity and prognosis of COVID-19 patients are still challenges for clinicians. The Sequential Organ Failure Assessment (SOFA) score is one of the scoring systems used to evaluate organ failure and can predict the severity and outcome of the disease.^[6,7]^ The SOFA scoring system was launched in 1996, and its performance is based on the evaluation of the following 6 major organ functions: circulation, respiration, liver, renal function, central nervous system, and coagulation function. The score of each organ is between 0 and 4. It is an easy-to-use tool for systematically and continuously evaluating organ functions during hospitalization.^[8]^ Raschke study showed that SOFA scores are not a good discriminator of probable mortality in patients with COVID-19 pneumonia requiring mechanical ventilation because the study was conducted in critically ill patients admitted to the ICU for treatment and requiring mechanical ventilation.^[9]^ However, our study had a broader population that included all patients with a confirmed diagnosis of COVID-19. Therefore, a retrospective study was conducted to evaluate the accuracy of the SOFA score in predicting the severity and prognosis of COVID-19.

## 2 Methods

### 2.1 Study population

This is a retrospective observational study involving 117 hospitalized patients with COVID-19, all of whom were from Jingzhou Central Hospital between January 16, 2020 and March 23, 2020. The study was approved by the Institutional Ethics Committee of Jingzhou Central Hospital, and the requirement for informed consent from the study participants was waived. All adult patients diagnosed with the coronavirus disease 2019 according to the WHO interim guidelines^[10]^ were screened for multidisciplinary diagnosis and treatment by infectious disease, respiratory and intensive care physicians. Patients who were discharged or died from January 16, 2019 (when the first patient was admitted to the hospital), to March 23, 2020, were included in the study. Clinical outcomes (such as death) of all patients within 60 days were recorded from January 16, 2020 to the last date of follow-up.

### 2.2 Data collection

Trained physicians collected patient epidemiological, demographic, and clinical and laboratory data through the electronic medical record system. The SOFA score was evaluated when the patient had an onset of disease or admission. The patients were classified into 2 groups: mild (including mild and moderate) group and severe (including severe and critical) group. The mild group exhibited mild clinical symptoms, no imaging manifestations of pneumonia. The moderate group had fever and respiratory symptoms, and imaging showed pneumonia. Patients in the severe group had dyspnea, with RR ≥30 beats/min at rest, average oxygen saturation ≤93%. Patients in the critical group had the occurrence of shock, respiratory failure requiring mechanical ventilation, and multiple organ failure requiring ICU monitoring and treatment.^[11]^

### 2.3 Procedures

Before admission, the nasopharyngeal swabs of all patients were routinely collected for SARS-CoV-2 real-time polymerase chain reaction detection, and patients with positive findings were admitted to the hospital. After admission, laboratory testing and imaging examination (computed tomography scan) were routinely performed in all patients. Routine examinations included liver and renal function, electrolytes, procalcitonin, myocardial zymogram, interleukin-6 (IL-6), complete blood count, blood gas analysis, high sensitivity C-reactive protein (hs-CRP), etc. Patients reached the discharge criteria when they had a normal body temperature for ≥3 days, their respiratory symptoms were relieved, the lung computed tomography manifestations significantly improved, and they had 2 consecutive nasopharyngeal swabs with negative results from nucleic acid tests (the sampling time was at least 24 hours apart).

### 2.4 Statistical analysis

Continuous variables with a normal distribution and nonnormal distribution are summarized as the mean ± standard deviation and the median (interquartile range [IQR]), respectively. Categorical variables are expressed as frequencies or percentages. Differences between severe and mild cases were analyzed using the χ^2^*test*, *Mann–Whitney U test* or *Fisher* exact *test*, where appropriate. Univariate and multivariate logistic regression models were used to identify the risk factors associated with the severity of COVID-19. All clinically important covariates and those with a significant correlation in the univariate analyses (*P* *<* *.15*) were included in the multivariate analysis. Survival curves were drawn and the differences in survival were assessed using the Kaplan–Meier method and log-rank test. The cutoff value of the SOFA score was determined by receiver operating characteristic (ROC) curve analysis. Based on the cutoff value of the SOFA score, the patients were classified into 2 groups. The hazard ratios (HRs) with 95% confidence intervals for the risk factors for death were determined using Cox proportional hazards regression models. The Cox proportional hazards model was used to analyze the potential risk factors associated with the SOFA score for in-hospital death. All statistical analyses were carried out with SPSS (IBM, version 25.0). Graphs were plotted using the GraphPad Prism 8.0 (San Diego, CA, USA). A *P* value of <.05 was considered statistically significant, and all tests were two-tailed.

## 3 Results

### 3.1 Demographic, baseline, and clinical characteristics of patients with COVID-19

**Figure 1:**
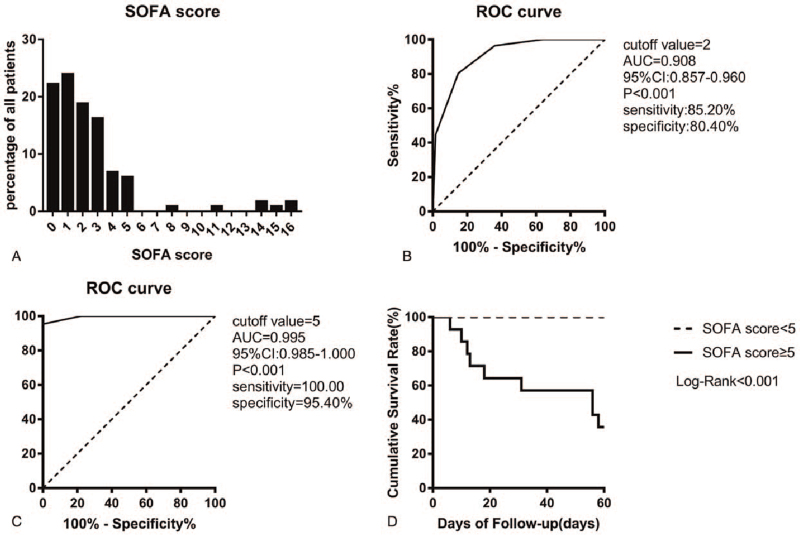
(A) Distribution of SOFA scores in patients with COVID-19. (B) ROC curves for patients with SOFA score = 2 in predicting the severity of COVID-19. (C) ROC curves for patients with SOFA score = 5 in predicting the death in the hospital. (D) Kaplan–Meier survival curves for patients with COVID-19 in the SOFA score ≥5 group and in the SOFA score <5 groups. COVID-19 = coronavirus disease 2019, ROC = receiver operating characteristic, SOFA score = Sequential Organ Failure Assessment score.

We analyzed 117 confirmed COVID-19 patients, including 60 males, accounting for 51.3%. More than half (61) of the patients were identified as critical patients, and 9 of them died. The majority of severe patients were men, accounting for 55.7%. The median age of the patients was 50.0 years (IQR, 35.5–62.0 years), ranging from 18 to 83 years. The median age of seriously ill patients was significantly higher than that of mild patients [56.0 (IQR, 45.0–68.0) vs 38.0 (IQR, 31.0–54.5)]. Sixty-three patients had comorbidities, and hypertension (27.4%) was the most common comorbidity, followed by diabetes (8.5%), stroke (4.3%), coronary heart disease (3.4%), chronic liver disease (CLD1) (3.4%), and so on. The proportion of hypertension in severe patients was higher than that in mild patients (42.6% vs 10.7%, *P* < .001). It is observed that on admission, fever (82.9%) and dry cough (70.1%) were the most common symptoms, followed by dyspnea (39.3%), fatigue (29.1%), and sore throat (9.4%). The median time from onset to the admission of all patients was 5 days (IQR, 3–7 days), no difference was found between the mild and severe group (Table 1). The median SOFA score of the patients was 2 (IQR, 1–3), the minimum SOFA score was 0, and the maximum SOFA score was 16. The distribution of SOFA scores is shown in Figure 1A. The median SOFA score of severe patients was higher than that of mild patients [3 (IQR, 2–4) vs 1 (IQR, 0–1); *P* < .001].

**Table 1:** Demographics and baseline characteristics of patients with COVID-19.

Demographics and clinical characteristics	All patients (n = 117)	Mild (n = 56)	Severe (n = 61)	*P* value
Age, yrs	50.0 (35.5–62.0)	38.0 (31.0–54.5)	56.0 (45.0–68.0)	<.001
Sex				.302
Male	60, 51.3%	26, 46.4%	34, 55.7%	
Female	57, 48.7%	30, 53.6%	27, 44.3%	
Comorbidity				
Hypertension	32, 27.4%	6, 10.7%	26, 42.6%	<.001
Diabetes	10, 8.5%	2, 3.6%	8, 13.1%	.058
Coronary heart disease	4, 3.4%	1, 1.8%	3, 4.9%	.352
Stroke	5, 4.3%	2, 3.6%	3, 4.9%	.655
Carcinoma	3, 2.6%	0	3, 4.9%	.093
Chronic liver disease	4, 3.4%	3, 5.4%	1, 1.6%	.269
Chronic kidney disease	1, 0.9%	0	1, 1.6%	.336
Chronic lung disease	2, 1.7%	0	2, 3.2%	.172
Other	2, 1.7%	0	2, 3.2%	.172
Signs and symptoms				
Fever	97, 82.9%	48, 85.7%	49, 80.3%	.919
Cough	82, 70.1%	35, 62.5%	47, 77.0%	.185
Dyspnea	46, 39.3%	8, 14.3%	38, 62.3%	<.001
Diarrhea	5, 4.3%	3, 5.4%	2, 3.3%	.655
Fatigue	34, 29.1%	18, 32.1%	16, 26.2%	.732
Sore throat	11, 9.4%	7, 12.5%	4, 6.6%	.366
Myalgia	5, 4.3%	3, 5.4%	2, 3.3%	.655
Sputum production	6, 5.1%	2, 3.6%	4, 6.6%	.414
Highest temperature (°C)	38.40 (37.75–38.80)	38.25 (37.70–38.70)	38.50 (37.80–39.00)	.058
SOFA score	2 (1–3)	1 (0–1)	3 (2–4)	<.001
Lowest SPO2 (%)	94 (90–95)	95 (94–95)	90 (86–92)	<.001
Days from symptoms to hospital admission	5 (3–7)	4 (3–7)	5 (3–7)	.867

### 3.2 Laboratory characteristics of the study population

Table 2 summarizes the laboratory test results of the patients after admission. The median lymphocyte count (LYM) of the patients was 0.860  × 10^9^/mL. The LYM in severe patients was lower than that in mild patients [0.640 (IQR, 0.420–0.965) vs 1.035 (IQR, 0.690–1.360); *P* < .001]. The hs-CRP, white blood cell (WBC), neutrophil (NE), and gamma-glutamyl transpeptidase levels in severe patients were higher than those in mild patients. In addition, the procalcitonin, direct bilirubin (DBIL), alanine aminotransferase (ALP), cystatin C, and creatine kinase muscle-brain isoform (CK-MB) levels of severe patients were higher than those of mild patients.

**Table 2:** Laboratory findings of patients with COVID-19.

Laboratory findings	Normal range	All patients (n = 117)	Mild (n = 56)	Severe (n = 61)	*P* value
Procalcitonin, ng/mL	0–0.5	0.060 (0.0350–0.110)	0.040 (0.030–0.060)	0.080 (0.050–0.165)	<.001
IL-6, pg/L	0–7	27.489 (8.685–27.489)	27.489 (7.247–27.489)	22.700 (8.685–34.290)	.224
White blood cells, × 10^9^/mL	3.5–9.5	7.300 (5.025–11.270)	6.185 (4.400–9.175)	9.990 (6.525–12.940)	<.001
Neutrophils, × 10^9^/mL	1.8–6.3	6.190 (3.460–9.910)	4.495 (2.872–7.247)	8.320 (4.925–11.900)	<.001
Lymphocytes, × 10^9^/mL	1.1–3.2	0.860 (0.560–1.215)	1.035 (0.690–1.360)	0.640 (0.420–0.965)	<.001
Monocytes, × 10^9^/mL	0.1–0.6	0.400 (0.300–0.530)	0.400 (0.292–0.497)	0.380 (0.305–0.535)	.733
Eosinophil, × 10^9^/mL	0.02–0.52	0.000 (0.000–0.055)	0.005 (0.000–0.067)	0.000 (0.000–0.045)	.233
Basophil, × 10^9^/mL	0–0.06	0.010 (0.010–0.030)	0.010 (0.010–0.020)	0.020 (0.010–0.035)	.114
Red blood cell, × 10^12^/mL	3.8–5.1	4.214 ± 0.623	4.339 ± 0.530	4.099 ± 0.683	.035
Hemoglobin, g/L	115–150	134.000 (118.500–145.000)	134.000 (121.000–146.750)	133.000 (116.000–141.500)	.125
Platelets, × 10^9^/mL	125–350	198.000 (157.500–259.000)	194.500 (156.000–231.500)	217.000 (159.000–279.000)	.200
hs-CRP, mg/L	0–10	8.760 (2.560–25.410)	4.815 (0.870–16.967)	16.830 (4.730–43.665)	<.001
Total bilirubin, umol/L	<21	11.400 (9.100–16.200)	11.000 (8.650–13.925)	13.000 (9.300–18.150)	.053
Direct bilirubin, umol/L	<7.5	3.700 (2.500–5.800)	3.200 (2.325–4.400)	4.300 (2.800–7.150)	.009
Indirect bilirubin, umol/L	<18.9	7.800 (6.250–10.600)	7.700 (6.000–9.875)	8.200 (6.500–11.250)	.180
Alanine aminotransferase, U/L	7–40	63.400 (28.700–117.450)	55.600 (22.050–104.475)	68.500 (33.700–127.500)	.167
Aspartate aminotransferase, U/L	13–35	33.200 (21.050–47.650)	28.200 (19.075–43.400)	36.700 (22.250–51.000)	.114
Alkaline phosphatase, U/L	40–150	54.800 (42.900–71.300)	53.750 (42.275–63.300)	59.200 (44.350–79.750)	.041
Gamma-glutamyl transpeptidase, U/L	7–45	54.600 (28.500–87.400)	40.700 (17.025–65.475)	65.200 (38.650–132.200)	<.001
Urea nitrogen, mmol/L	2.6–7.5	5.140 (4.130–6.515)	4.620 (3.790–5.520)	5.540 (4.645–7.665)	<.001
Creatinine, umol/L	41–73	60.800 (49.050–67.700)	61.250 (49.250–68.175)	60.600 (48.200–66.900)	.631
Uric acid, umol/L	142–339	238.740 ± 92.149	264.960 ± 81.093	214.660 ± 95.694	.003
Cystatin C, mg/L	0.54–1.15	0.870 (0.750–1.030)	0.820 (0.712–0.947)	0.960 (0.820–1.110)	<.001
Glomerular filtration rate, mL/min/	>90	130.500 (107.350–153.050)	129.250 (114.125–148.875)	132.000 (103.000–161.450)	.849
Potassium, mmol/L	3.5–5.3	4.340 ± 0.600	4.431 ± 0.575	4.257 ± 0.615	.119
Sodium, mmol/L	137.0–147.0	140.182 ± 3.315	141.094 ± 2.592	139.344 ± 3.368	.002
Chlorine, mmol/L	99.0–110.0	103.800 (101.100–105.400)	104.350 (102.425–105.775)	102.200 (100.600–104.800)	.006
Calcium, mmol/L	2.11–2.52	2.000 (1.900–2.155)	2.025 (1.940–2.187)	1.980 (1.850–2.145)	.122
Magnesium, mmol/L	0.70–1.15	0.986 ± 0.093	0.988 ± 0.093	0.984 ± 0.093	.857
Phosphorus, mmol/L	0.85–1.51	1.107 ± 0.247	1.183 ± 0.185	1.036 ± 0.277	.001
CK, U/L	<167	73.800 (48.500–109.925)	74.600 (45.175–105.331)	72.900 (49.400–124.000)	.561
CK-MB, U/L	<24	14.400 (10.500–18.775)	13.500 (9.750–16.312)	16.900 (12.900–23.300)	.001
cTnI, ug/L	<0.04	0.010 (0.010–0.040)	0.010 (0.010–0.035)	0.010 (0.010–0.042)	.579
Myoglobin, ug/L	1.5–70.0	31.000 (18.900–54.750)	27.250 (17.475–42.600)	33.650 (24.300–55.500)	.069

### 3.3 Analysis of risk factors for severe COVID-19

To determine the risk factors for the bad prognosis of patients with severe disease, we compared the clinical and laboratory characteristics of mild and severe patients. Univariate logistic regression analysis showed that age, hypertension, SOFA score, and the levels of IL-6, WBC, NE, LYM, red blood cell, hs-CRP, DBIL, ALP, GGT, urea nitrogen, uric acid, cystatin C, sodium, chlorine, phosphorus and CK-MB were related to the aggravation of the patient's condition. Multivariable logistic regression analysis showed that SOFA score, advanced age, and hypertension were independently associated with the risk of severe COVID-19. In addition, the levels of WBC, NE, LYM, ALP, phosporous, urea nitrogen, cystatin C and CK-MB were also related to severe COVID-19. Univariate analysis showed that SOFA score was a risk factor for patients with severe COVID-19, with an odds ratio of 5.328 (95% CI: 2.932–9.681; *P* < .001). Multivariate analysis also revealed that SOFA score was a risk factor for patients with severe COVID-19 (OR = 5.851; 95% CI: 3.044–11.245; *P* *<* *.001*) (Table 3).

**Table 3:** Risk factors associated with severe COVID-19 patients.

Risk factors	Univariable OR (95% CI)	*P* value	Multivariable OR (95% CI)	*P* value
Age, years	1.064 (1.034–1.094)	.000	1.069 (1.036–1.103)	<.001
Hypertension	6.190 (2.307–16.614)	.000	7.310 (1.705–31.350)	.007
Diabetes	4.075 (0.827–20.088)	.084		
SOFA score	5.328 (2.932–9.681)	.000	5.851 (3.044–11.245)	<.001
IL-6	1.021 (1.002–1.041)	.029		
White blood cells	1.196 (1.078–1.327)	.001	1.195 (1.060–1.346)	.004
Neutrophils	1.232 (1.107–1.371)	.000	1.210 (1.084–1.351)	.001
Lymphocytes	0.222 (0.094–0.526)	.001	0.280 (0.107–0.730)	.009
Red blood cell	0.520 (0.278–0.973)	.041		
hs-CRP	1.026 (1.008–1.045)	.006		
Direct bilirubin	1.207 (1.016–1.434)	.032		
Alkaline phosphatase	1.020 (1.002–1.039)	.032	1.032 (1.007–1.057)	.013
Gamma-glutamyl transpeptidase	1.006 (1.001–1.012)	.027		
Urea nitrogen	1.484 (1.173–1.876)	.001	1.480 (1.154–1.898)	.002
Uric acid	0.993 (0.989–0.998)	.005		
Cystatin C	33.239 (4.316–255.970)	.001	30.893 (2.988–319.403)	.004
Sodium	0.819 (0.715–0.937)	.004		
Chlorine	0.844 (0.743–0.960)	.010		
Phosphorus	0.068 (0.012–0.380)	.002	0.055 (0.007–0.462)	.008
CK-MB	1.092 (1.027–1.161)	.005	1.086 (1.017–1.161)	.030
Procalcitonin	5.689 (0.710–45.575)	.102		
Hemoglobin	0.980 (0.960–1.000)	.056		
Platelets	1.004 (0.999–1.009)	.096		
Total bilirubin	1.057 (0.995–1.123)	.072		
Indirect bilirubin	1.068 (0.981–1.163)	.128		
Potassium	0.608 (0.325–1.139)	.120		
Calcium	0.220 (0.032–1.485)	.120		
Myoglobin	1.004 (0.999–1.009)	.112		

### 3.4 ROC curves and cumulative survival curves for predicting the severity and prognosis of COVID-19

ROC curves were constructed to evaluate the predictive value of the SOFA score for the severity and prognosis of COVID-19 (Fig. 1B and C). The area under the receiver operating characteristic curve (AUC) was used to evaluate the diagnostic accuracy of the SOFA score for predicting severe COVID-19 (cutoff value = 2; AUC = 0.908 [95% CI: 0.857–0.960]; sensitivity: 85.20%; specificity: 80.40%) and the risk of death in COVID-19 patients (cutoff value = 5; AUC = 0.995 [95% CI: 0.985–1.000]; sensitivity: 100.00%; specificity: 95.40%). Kaplan–Meier curve analysis and the log-rank test were performed to assess the cumulative survival rates and compare the 60-day survival curves between the high SOFA score group (SOFA score ≥5) and the low SOFA score group (SOFA score <5). Patients in the high SOFA score group had a significantly higher risk of death than those in the low SOFA score group (*log-rank*, *P* < .001) (Fig. 1D).

### 3.5 Results of cox proportional hazards regression analysis

**Figure 2:**
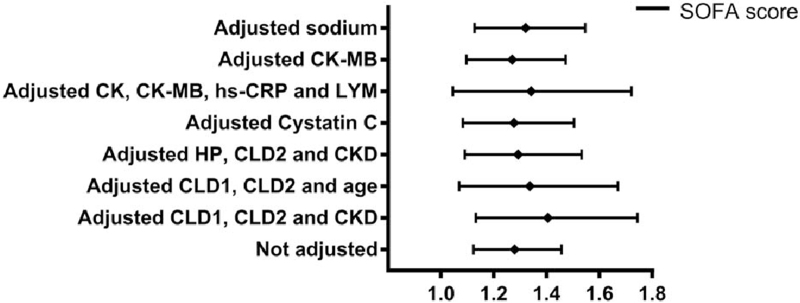
Forest plots demonstrating the association of SOFA score with the death of COVID-19 patients in the hospital. CK = creatine kinase, CKD = chronic kidney disease, CK-MB = creatine kinase muscle-brain isoform, CLD1 = chronic liver disease, CLD2 = chronic lung disease, COVID-19 = coronavirus disease 2019, HP = hypertension, hs-CRP = high-sensitivity C-reactive protein, LYM = lymphocytes, SOFA score = Sequential Organ Failure Assessment score.

Cox proportional hazards regression analysis was used to assess the potential association between the SOFA score and hospital death. The univariate analysis indicated that the SOFA score was associated with a higher risk of hospital death (HR = 1.279, 95% CI: 1.123–1.456, *P* < .001). In addition, patient age, lowest oxygen saturation (SPO2), hypertension, CLD1, chronic lung disease (CLD2), chronic kidney disease (CKD), and the levels of red blood cell, hemoglobin, hs-CRP, total bilirubin (TBIL), DBIL, aspartate aminotransferase, ALP, urea nitrogen, creatinine, cystatin C, cardiac troponin I (cTnI), and myoglobin were associated with the risk of death in the hospital (Table 4). Multivariate Cox proportional hazards regression analysis was used to evaluate the independent prognostic effect of the SOFA score. After adjusting for CLD2, CKD, and CLD1 (model 1), the HR of the SOFA score for predicting hospital deaths was 1.405 (95% CI: 1.132–1.744, *P* *=* *.002*). After adjusting for age, CLD1, and CLD2 (model 2), the HR was 1.336 (95% CI: 1.069–1.670, *P* *=* *.011*). After adjusting for hypertension, CLD2, and CKD (model 3), the HR was 1.292 (95% CI: 1.090–1.532, *P* *=* *.003*). After adjusting for cystatin C (model 4), the HR was 1.276 (95% CI: 1.083–1.504, *P* *=* *.004*). After adjusting for LYM, hs-CRP, creatine kinase (CK), and CK-MB (model 5), the HR was 1.341 (95% CI: 1.045–1.721, *P* *=* *.021*). After adjusting for CK-MB (model 6), the HR was 1.270 (95% CI: 1.096–1.472, *P* *=* *.001*). After adjusting for Na (model 7), the HR was 1.320 (95% CI: 1.127–1.546, *P* *=* *0.001*). In this process, age, CLD1, CKD, CLD2, cystatin C, hs-CRP, CK, and CK-MB also showed significance for independently predicting hospital death, while Na showed a protective effect (Table 5). Forest plots depicting the results of the multivariate analysis of each SOFA score model assessed by the Cox proportional hazards regression model are shown in Figure 2.

**Table 4:** Results of univariate Cox proportional-hazards regression analyzing the effect of variables on in hospital death.

Risk factors	HR (95%CI)	*P* value
Age	1.101 (1.026–1.181)	.007
SOFA score	1.279 (1.123–1.456)	<.001
Lowest SPO2	0.910 (0.872–0.950)	<.001
Hypertension	6.083 (1.173–31.540)	.032
Chronic lung disease	13.079 (1.450–117.959)	.022
Chronic kidney disease	24.937 (2.768–224.666)	.004
Chronic liver disease	12.467 (1.270–122.355)	.030
Red blood cell	0.141 (0.045–0.441)	.001
Hemoglobin	0.942 (0.909–0.976)	.001
hs-CRP	1.020 (1.004–1.037)	.015
Total bilirubin	1.022 (1.004–1.041)	.019
Direct bilirubin	1.034 (1.008–1.062)	.010
Aspartate aminotransferase	1.006 (1.002–1.011)	.005
Alkaline phosphatase	1.007 (1.002–1.011)	.002
Urea nitrogen	1.318 (1.172–1.483)	<.001
Creatinine	1.006 (1.003–1.009)	<.001
Cystatin C	2.400 (1.618–3.558)	<.001
Sodium	0.779 (0.626–0.969)	.025
CK	1.007 (1.003–1.011)	<.001
CK-MB	1.040 (1.017–1.064)	.001
cTnI	1.072 (1.022–1.125)	.004
Myoglobin	1.007 (1.004–1.010)	<.001
Highest temperature	0.449 (0.194–1.041)	.062
Fever	0.212 (0.043–1.051)	.058
Dyspnea	6.088 (0.692–53.598)	.104
Lymphocytes	0.128 (0.009–1.904)	.136
Eosinophil	55.502 (0.613–5029.250)	.081
Platelets	0.990 (0.978–1.002)	.103
Indirect bilirubin	1.060 (0.997–1.127)	.064

**Table 5:** Results of multivariate Cox proportional-hazards regression analyzing the effect of variables on in hospital death.

Risk factors	HR (95%CI)	*P* value
Not Adjusted SOFA score	1.279 (1.123–1.456)	<.001
Mode 1		
SOFA score	1.405 (1.132–1.744)	.002
Chronic lung disease	93.516 (4.063–2152.432)	.005
Chronic kidney disease	10.216 (0.957–109.050)	.054
Chronic liver disease	69.136 (3.226–1481.626)	.007
Mode 2		
SOFA score	1.336 (1.069–1.670)	.011
Age	1.152 (1.018–1.302)	.024
Chronic lung disease	43.406 (1.869–1008.004)	.019
Chronic liver disease	1515.310 (10.430–220156.492)	.004
Mode 3		
SOFA score	1.292 (1.090–1.532)	.003
Hypertension	5.995 (0.655–54.887)	.113
Chronic lung disease	16.956 (1.197–240.105)	.036
Chronic kidney disease	21.147 (1.224–365.495)	.036
Mode 4		
SOFA score	1.276 (1.083–1.504)	.004
Cystatin C	1.913 (1.304–2.806)	.001
Mode 5		
SOFA score	1.341 (1.045–1.721)	.021
Lymphocytes	22.140 (0.756–647.954)	.072
hs-CRP	1.035 (1.001–1.071)	.043
CK	1.007 (1.002–1.012)	.009
CK-MB	1.055 (1.006–1.107)	.027
Mode 6		
SOFA score	1.270 (1.096–1.472)	.001
CK-MB	1.034 (1.007–1.061)	.014
Mode 7		
SOFA score	1.320 (1.127–1.546)	.001
Sodium	0.775 (0.623–0.964)	.022

## 4 Discussion

Severe COVID-19 can easily cause acute respiratory distress syndrome, multiple organ dysfunction, acute heart injury, acute kidney injury, and even death.^[4]^ Identifying the death risk associated with critically ill patients early and giving these patients priority treatment in a timely manner is particularly important in global health emergencies. Studies have shown that a total of 60 predictors can assess the severity of COVID-19, of which 7 factors are considered to be highly correlated and consistent, including SOFA score, age, d-dimer, hs-CRP, body temperature, albumin, and diabetes.^[12]^ The results of this study revealed that SOFA score, age, CKD, CLD1, CLD2, cystatin C, hs-CRP, CK, CK-MB, and other factors were independent risk factors for in-hospital death, which was similar to the results of the above study.

Zhou et al showed that older age, higher d-dimer levels, and higher SOFA scores in COVID-19 patients at admission were associated with high in-hospital mortality. In addition, increased levels of cTnI, lactate dehydrogenase, and lymphocytopenia were more common in patients with severe COVID-19.^[13]^ Also, studies have shown that SARS-CoV-2 can participate in and induce the activation of the complement and coagulation system, which is related to the severity of COVID-19 patients.^[14]^ Myocardial injury was another independent risk factor for deterioration and death in patients with COVID-19. The risk of death of hospitalized patients with myocardial injury was 6.6 to 26.9 times higher than that of patients without myocardial injury.^[15]^

In this study, the median age of seriously ill patients was 56.0 years, which was significantly higher than that of mild patients (38 years). Hypertension was the most common complication of COVID-19, especially in severe patients. A review of the literature showed that COVID-19 patients with hypertension, especially elderly patients, had a 2.5-fold increased risk of serious or even fatal events.^[16]^ Another large cohort study showed that in addition to some factors, such as advanced age, male, asthma, diabetes, increased risk of death in COVID-19 patients, poverty, and ethnicity (African Americans and South Asians) were also associated with the death of COVID-19 patients.^[17]^

At present, published studies have not systematically evaluated the accuracy of the SOFA score in the diagnosis of COVID-19 severity and its predictive value. The SOFA score was originally used to assess the severity of organ dysfunction in patients with severe sepsis and has been validated in ICU patients in multiple regions.^[18]^ As critically ill patients usually have multiple organ dysfunction, the SOFA score has been widely used to predict the clinical outcomes of critically ill patients, such as predicting mortality in patients with chronic liver failure and hematological malignancies.^[19,20]^ Gupta et al summarized the clinical characteristics of SARS-CoV-2 infection, which could not only cause severe lung injury but also damaged the heart, liver, kidney, nervous system, endocrine system, blood system, and skin, resulting in arrhythmia, acute coronary syndrome, thrombosis, gastrointestinal symptoms, hyperglycemia, and skin rash.^[21]^ Thus, the SOFA score can comprehensively assess multiple organ dysfunction caused by SARS-CoV-2.

In our study, the SOFA score was also recognized as a valuable prognostic tool for the outcome of patients with COVID-19. Univariate regression analysis showed that the increase in SOFA score and IL-6 and the decrease in lymphocyte count were related to the aggravation of the patient's condition. Multivariable regression analysis demonstrated that SOFA score, advanced age, and hypertension were independently associated with the risk of severe COVID-19. At present, it is believed that COVID-19 leads to organ failure, which is mainly related to cytokine storm and immunosuppression; the clinical manifestations are persistent fever, hemocytopenia, and organ involvement.^[22]^ The laboratory results were characterized by increased levels of inflammatory factors such as granulocyte colony-stimulating factor, interleukin-2 (IL-2), IL-6, interleukin-7 (IL-7), interferon-γ-inducible protein-10 (IP-10), tumor necrosis factor α, macrophage inflammatory protein-1 α and monocyte chemoattractant protein 1.^[23,24]^ The analysis of the immune system of patients with severe COVID-19 showed that the number of innate immune cells increased, while T cells decreased. In COVID-19 patients, the early increase of cytokines was positively correlated with poor prognosis.^[25]^ This may well explain why dexamethasone and tocilizumab have been found to reduce mortality in many clinical trials for COIVD-19.^[26,27]^ Therefore, the SOFA score can reflect not only multiple organ failure but also the degree of inflammation and can accurately predict the severity of the patient's disease.

Sepsis is life-threatening organ dysfunction, caused by the dysregulated host response to infection. Rapid change in SOFA score ≥2 points after infection is regarded as the clinical criterion of sepsis-associated organ dysfunction. The SOFA score ≥2 reflects approximately 10% of the overall risk of death of suspected infected patients in general hospitals, and even patients with moderate organ dysfunction may further deteriorate. Therefore, it emphasizes the seriousness of this situation and reminds clinicians to intervene in a timely and appropriate manner.^[28]^ In this study, the AUC of the SOFA score was 0.908 (95% CI: 0.857–0.960) with a diagnostic cut-off value of 2 and a sensitivity and specificity of 85.20% and 80.40%, respectively. This result suggests that a SOFA score ≥2 can predict the severity of COVID-19 patients. Another study also showed that among 184,875 patients admitted to the ICU, an increase of 2 or more in the SOFA score had greater prognostic accuracy for in-hospital mortality than quick SOFA score or the systemic inflammatory response syndrome standard.^[29]^ When the cutoff value of the optimal SOFA score is 5 (AUC: 0.995, 95% CI: 0.985–1.000, sensitivity: 100.00%, specificity: 95.40%), the risk of mortality in patients with COVID-19 can be predicted. Regarding the 60-day mortality rate of patients in the high and low SOFA score groups, patients in the high SOFA score group (SOFA score ≥5) had a significantly higher risk of death than those in the low SOFA score group (SOFA score < 5). Wang et al used the SOFA score to assess the predictive value of early sepsis and 30-day mortality after liver transplantation, indicating that the survival rate of patients with SOFA score >5 within 1–7 days after liver transplantation was significantly lower than that of patients with SOFA score ≤5.^[30]^ Therefore, SOFA score ≥5 can be used as a good predictor of hospital mortality in COVID-19 patients. In addition, univariate and multivariate Cox proportional hazards regression analyses demonstrated that there was a high correlation between the SOFA score and hospital mortality, and the SOFA score was a risk factor for death in COVID-19 patients. These results provide strong evidence for priority in treatment and early special care for patients.

### 4.1 Study limitations

Nevertheless, some limitations should be considered when interpreting the results of this study. First, this is a single-center retrospective study involving a relatively small number of patients. Second, our study was limited by its retroactive design, which resulted in some data being unavailable in the electronic medical records. In some cases, if the patient's condition was stable during hospitalization without dyspnea and hypoxia, blood gas analysis was not performed, so the SOFA score could not be calculated accurately and had to be estimated by the Expectation-Maximization algorithm. However, in our study, the data loss rate of this variable was less than 25%. Finally, the retrospective nature of our study may lead to selection bias, and the findings need to be verified and refined by future prospective studies.

## 5 Conclusions

At present, the world is in the midst of a pandemic of COVID-19, making it a serious public health threat on a global scale. COVID-19 can lead to serious illness and death. Therefore, early identification and prediction of COVID-19 disease progression are critical. Given this background, a simple and practical tool for predicting the prognosis of patients with COVID-19 is particularly important. Our study suggests that SOFA scores may be an independent risk factor for hospital death and can be used well to assess the severity and prognosis of COVID-19.

## Acknowledgments

We thank all patients and medical staff at Jingzhou Central Hospital who were involved in this study.

## Author contributions

Zheng Yang, Qinming Hu, and Yi Sun conceived the study idea, and performed interpretation, manuscript writing, and final approval. Fei Huang and Shouxin Xiong performed data analysis and collection. All authors reviewed and approved the final version of the manuscript.

**Conceptualization:** Zheng Yang, Qinming Hu, Yi Sun.

**Data curation:** Fei Huang, Shouxin Xiong.

**Formal analysis:** Fei Huang, Shouxin Xiong.

**Funding acquisition:** Yi Sun.

**Investigation:** Zheng Yang, Qinming Hu, Yi Sun.

**Methodology:** Fei Huang, Shouxin Xiong.

**Writing – original draft:** Zheng Yang, Qinming Hu, Yi Sun.

**Writing – review & editing:** Zheng Yang, Qinming Hu, Yi Sun.
